# inSPOT: The First Online STD Partner Notification System Using Electronic Postcards

**DOI:** 10.1371/journal.pmed.0050213

**Published:** 2008-10-21

**Authors:** Deb Levine, Andrew J Woodruff, A. Rain Mocello, Jaime Lebrija, Jeffrey D Klausner

## Abstract

Deb Levine and colleagues describe an innovative online e-card service for partner notification, initial evaluation results, and future research needs.

In the United States there are 19 million new sexually transmitted disease (STD) cases diagnosed each year, including 900,000 reported cases of chlamydia, 330,000 reported cases of gonorrhea [1,2], and 55,400 estimated new HIV infections per year [3]. Notifying sexual partners of their potential exposure to an STD has been a mainstay of disease prevention and control since the 1930s [4]. Recent evidence-based reviews concluded that partner notification is effective for identifying those at risk for STDs [5] and HIV infection [6].

Traditionally, partner notification has been done in person, by phone, or by mail, with the assistance of a public health investigator. The high number of cases of gonorrhea and chlamydia, however, makes partner notification for all named partners impractical in many jurisdictions [7]. Particularly among gay men and other men who have sex with men (G/MSM), who tend to have higher numbers of partners, online notification may be an effective strategy to increase partner notification [8]. Recent survey data suggest that with the ease and privacy of online communication, more patients would be willing to receive notification of possible exposure to disease via e-mail or other new technologies [9]. Other studies have shown that online partner notification is an efficient method for reaching individuals otherwise inaccessible [10,11]. In a sample of 833 G/MSM in San Francisco, California, 73% responded “yes” when asked, “If you were diagnosed with an STD, would you consider sending an anonymous e-card to notify anyone you had sex with?” The primary reason for being unwilling to use an e-card to notify a partner was the respondent's preference to notify his partner/s in person [12]. This article will describe an innovative online e-card service for partner notification, the process of replication of this site across cities, states, and countries, initial evaluation results, and future research needs.

“For some partners, all I had was an email address. After sending inSPOT cards, I got some backlash, but the ending was always, ‘I'm glad you told me.’” — Male, CA

In 2004, the San Francisco Department of Public Health (SFDPH), and Internet Sexuality Information Services (ISIS), a 501(c)3 nonprofit organization, conducted needs assessment and community discussions with G/MSM in San Francisco and determined that most men told their primary partners when they were diagnosed with an STD (either by themselves or with the help of a public health investigator), but despite good intentions, they did not tell their casual sex partners. Men overwhelmingly said that if there were an easy, convenient, and anonymous way to inform their partners of their potential disease exposure, they would use it. ISIS then developed inSPOT (http://www.inSPOT.org/), a peer-to-peer, Web-based, STD partner notification system, in partnership with SFDPH and a local community advisory board. inSPOT is a Web site that uses electronic postcards (e-cards) to assist people in disclosing an STD diagnosis to their sex partner(s).

## Program Description

inSPOT was originally targeted to G/MSM because surveillance data showed that this population used the Internet increasingly to meet sex partners, and such partnering was associated with increases in disease transmission [13–15]. In 2005 and 2006, ISIS conducted six focus groups in Philadelphia, Pennsylvania; Indianapolis, Indiana; and San Francisco, California with a variety of people to inform the expansion of the service for heterosexual audiences. Participants agreed that all sexually active people could benefit from this service, regardless of the gender of their sex partners. The site was subsequently updated in April 2006 for all audiences.

The site design was based on extensive input from key community advisors and on usability testing in San Francisco with repeated samples of the general population. Consequently, inSPOT is very simple. The two sections are *Tell Them* and *Get Checked*. In *Tell Them*, users follow this path:

- Choose one of six e-cards (Figure 1),
- Type in recipients' e-mail addresses (up to six),
- Select an STD from a pull-down menu,
- Type in own e-mail address or send anonymously,
- Type in an optional personal message.

**Figure 1:**
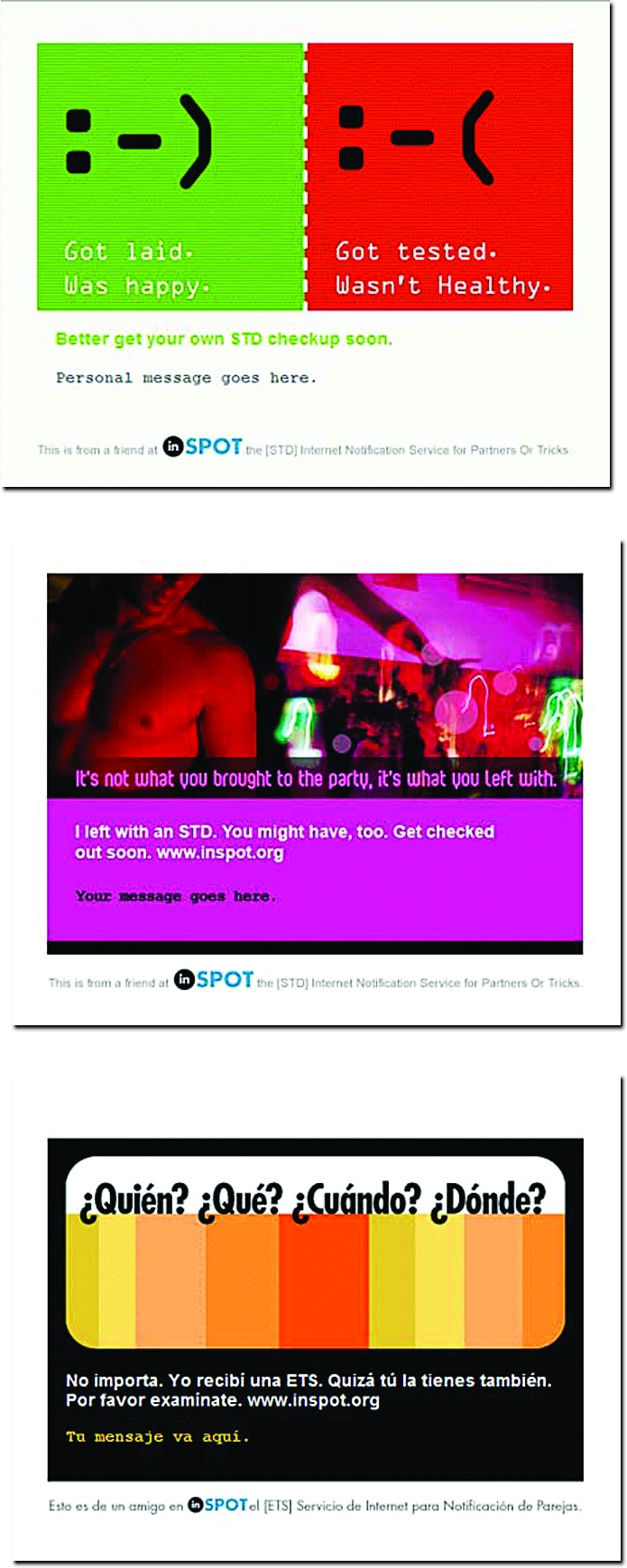
Examples of E-Cards

When an e-card is clicked on by the recipient, users are linked to a page with disease-specific information.

The *Get Checked* section is divided into STD information, a map of local testing sites, and links to online resources. To ensure the privacy of the user, no database to store e-mail addresses or information about e-card senders or recipients exists.

Since its 2004 launch in San Francisco, inSPOT has been replicated in three countries, ten cities, and nine states. The SFDPH bore the initial development costs of the site, estimated at US$50,000. The City and County of Los Angeles, California paid approximately US$16,000 for the first site replication, which ISIS created manually, running significantly over budget. Once interest was generated, ISIS hired an engineering firm to create a content management tool to make future replications easier, with fixed costs now set at about US$15,000, and annual maintenance fees of about US$3,000. Various local jurisdictions have additional modifications on their sites, such as banner ads (Portland, Oregon), electronic antibiotic prescriptions available to notified contacts of chlamydia or gonorrhea as expedited partner therapy [16] (San Francisco, CA), and second languages (Ottawa, Canada).

Each jurisdiction is encouraged to market inSPOT locally using palm cards and Internet banner advertisements created by ISIS. inSPOT San Francisco relied on placement of one billboard; palm card distribution to clinics and providers and at local events; and word-of-mouth marketing among residents.

New locations are added to the main inSPOT portal page (the landing page that includes links to each participating jurisdiction) in the following manner:

- A local jurisdiction or community organization contracts with ISIS, and provides information about local testing and treatment services.
- ISIS confirms information about local resources, testing sites, etc.
- ISIS creates a local map of the region, finds clinic locations on the map, and parses information into regional subdivisions.
- ISIS builds local inSPOT and provides online access to the sample site for review and approval for placement on the portal at http://www.inSPOT.org/.

To keep inSPOT e-cards out of e-mail spam filters, continuous testing and updating of the subject line of the e-card messages has been necessary. Obtaining and maintaining current clinic hours and services for each participating jurisdiction requires regular communication between ISIS and participating jurisdictions, and a regular schedule of phone calls confirming clinic information in each location every six to 12 months.

## Evaluation

**Number of cards sent.** Over 750 people visit the inSPOT.org portal daily. Since its launch in 2004, more than 30,000 people have sent over 49,500 e-cards. While we prepared for the possibility of misuse of the site by people sending e-cards maliciously, fewer than ten recipients have reported receiving an e-card in error.

The number of e-cards sent from all sites in 2006 totaled 16,983 and ranged from 280 in Portland to 9,916 in Los Angeles. In 2007, an additional two cities and three states began using inSPOT and a total of 6,622 e-cards were sent, with Idaho logging the lowest usage (45 e-cards) and Los Angeles again showing the highest usage (2,782 e-cards), albeit significantly lower than seen in the previous year. The overall average number of recipients per card was 1.6 and ranged from 1.2 to 2.0.

**Pattern of diseases.** In 2006 and 2007, 23,594 e-cards were sent, 3,631 (15.4%) for gonorrhea, 3,519 (14.9%) for syphilis, 2,203 (9.3%) for HIV, 2,736 (11.6%) for chlamydia, and 11,505 (48.8%) for any “Other” STDs, including cervicitis; “crabs”; scabies; hepatitis A, B, and C; *Lymphogranuloma venereum*; *Molluscum contagiosum*; nongonococcal urethritis; *Shigella*; trichomoniasis; and “Unspecified” (Figure 2).

**Figure 2:**
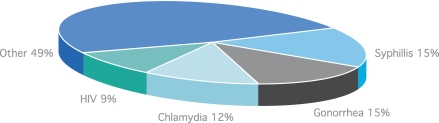
E-Cards Sent, By Disease, 2006–2007

Although primary, secondary, and early latent syphilis accounted for 1.3% of nationally reportable STDs in 2006 (i.e., gonorrhea, chlamydia, and syphilis, excluding congenital and late latent syphilis), a much greater proportion of e-cards (9.4%) sent during that same period notified recipients about potential syphilis exposure. In contrast, chlamydia represented 73.2% of all nationally reportable STDs, and only 9.2% of e-cards were sent for that disease. This discrepancy suggests that the population of inSPOT users may be different from the US general population or that inSPOT users selectively choose for which STDs to use inSPOT's partner notification services. Additionally, awareness and education efforts about the importance of partner notification included in syphilis prevention in major US cities since the late 1990s could have influenced the overall number of syphilis e-cards sent [17]. In particular, online partner notification became crucial when syphilis outbreaks began to be linked to online venues [18].

**“Click-through” rates.** Finally, we analyzed rates at which e-card recipients clicked a link embedded in the card that connected to STD test site information. Annual “click-through” rates ranged from 20.4% in Los Angeles to 48.2% in Idaho, with an average across all sites of 26.8% in 2006 and 28.5% in 2007. During the period that inSPOT has been active, from December 2005 through February 2008, 29,137 people accessed STD testing information as a result of receiving an e-card. In addition, surveys of the general population and HIV providers in San Francisco indicated awareness and acceptance of the service (Table 1).

**Table 1:**
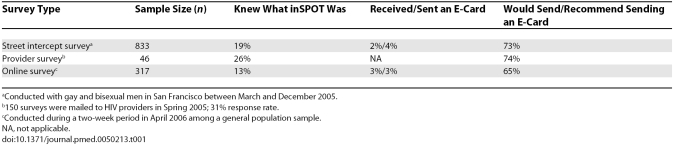
Summary of Various Samples of inSPOT Awareness, 2005–2006

## inSPOT and Public Health Investigators

While inSPOT was never intended to replace traditional partner notification by public health investigators, it has emerged as a complement to those services.

*“We interviewed a guy who was diagnosed with secondary syphilis. He had received an anonymous card through InSPOT informing him that he had been exposed.”*

*“Our patient's partner got an anonymous notification through inSPOT that he had been exposed to syphilis and contacted our patient. Our patient immediately went to the clinic website for information, got tested thru the clinic's online testing program, and came to City Clinic for treatment. The power of the Internet age…”* — Public Health Investigators in San Francisco, CA

## Next Steps/Discussion

inSPOT has the potential to be a national and international resource. In countries that lack extensive technology infrastructure, people access the Web in public Internet cafes [19] and, increasingly, via their portable digital assistants and cell phones. In the US, further Web site growth has been hampered by the absence of a usable, current national database of public and private STD testing sites. ISIS is currently coordinating efforts across multiple national agencies to complete the inSPOT portal for all 50 states (Figure 3). inSPOT has been translated into Romanian and French and is in the process of being translated into Spanish.

**Figure 3:**
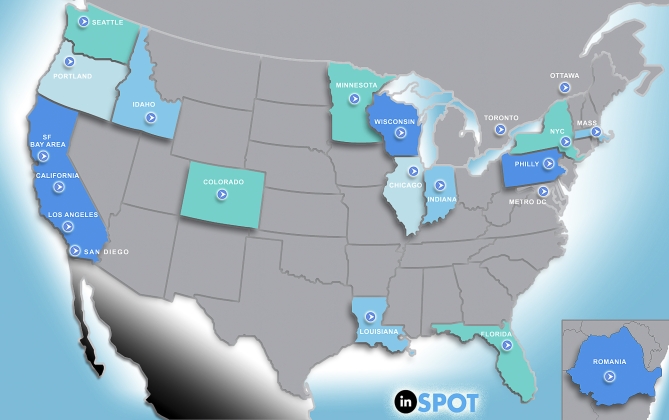
Currently Participating Jurisdictions

While we report usage data, ISIS does not have data on the proportion of site users who ultimately access STD testing as a direct result of receiving an e-card. We also do not have specific data linking site users to STD diagnoses. Future research efforts should be directed towards comprehensive evaluation to establish the effectiveness of inSPOT in increasing STD partner notification and testing and to assess its impact on reducing disease transmission.

## Abbreviations

ISIS: Internet Sexuality Information Services
G/MSM: gay men and other men who have sex with men
SFDPH: San Francisco Department of Public Health
STD: sexually transmitted disease

## References

[pmed-0050213-b001] Weinstock H, Berman S, Cates W (2004). Sexually transmitted diseases among American youth: Incidence and prevalence estimates, 2000. Perspect Sex Reprod Health.

[pmed-0050213-b002] Centers for Disease Control and Prevention (2006). Sexually transmitted disease surveillance 2005. http://www.cdc.gov/std/stats05/default.htm.

[pmed-0050213-b003] Hall HI, Ruiguang S, Rhodes P, Prejean J, An Q (2008). Estimation of HIV incidence in the United States. JAMA.

[pmed-0050213-b004] Parran T (1937). Shadow on the land.

[pmed-0050213-b005] Macke BA, Maher JE (1999). Partner notification in the United States: An evidence-based review. Am J Prev Med.

[pmed-0050213-b006] Hogben M, McNally T, McPheeters M, Hutchinson AB (2007). The effectiveness of HIV partner counseling and referral services in increasing identification of HIV-positive individuals: A systematic review. Am J Prev Med.

[pmed-0050213-b007] Hogben M, Kissinger P (2008). A review of partner notification for sex partners of men infected with chlamydia. Sex Transm Dis.

[pmed-0050213-b008] Menza TW, St. De Lore J, Fleming M, Golden MR (2008). Partner notification for gonococcal and chlamydial infections in men who have sex with men: Success is underestimated by traditional disposition codes. Sex Transm Dis.

[pmed-0050213-b009] Mimiaga MJ, Tefu AM, Gortmaker S, Koenen KC, Fair AD (2008). HIV and STD status among MSM and attitudes about internet partner notification for STD exposure. Sex Transm Dis.

[pmed-0050213-b010] Centers for Disease Control and Prevention (2004). Using the Internet for partner notification of sexually transmitted diseases—Los Angeles County, California, 2003. MMWR Morb Mortal Wkly Rep.

[pmed-0050213-b011] Vest JR, Valadez AM, Hanner A, Lee JH, Harris PB (2007). Using e-mail to notify pseudonymous e-mail sexual partners. Sex Transm Dis.

[pmed-0050213-b012] Levine D, Klausner JD, Kent C, Ahrens K, Scott K (2006). inSPOT.org: A unique online partner notification system. http://cdc.confex.com/cdc/std2006/techprogram/P10691.HTM.

[pmed-0050213-b013] Ashton M, Sopwith W, Clark P, McKevley D, Lighton L (2003). An outbreak no longer: Factors contributing to the return of syphilis in Greater Manchester. Sex Transm Infect.

[pmed-0050213-b014] Kim AA, Kent C, McFarland W, Klausner JD (2001). Cruising on the Internet highway. JAIDS.

[pmed-0050213-b015] McFarlane M, Bull SS, Rietmeijer CA (2002). Young adults on the Internet: Risk behaviors for sexually transmitted diseases and HIV. J Adolesc Health.

[pmed-0050213-b016] Klausner JD, McCright J, Levine D (2008). 21st century STD prevention and control: Empowering the community with internet-based tools, San Francisco, 2007. http://cdc.confex.com/cdc/std2008/techprogram/P15101.HTM.

[pmed-0050213-b017] McFarlane M, Kachur R, Klausner JD, Roland E, Cohen M (2005). Internet-based health promotion and disease control in the 8 cities: Successes, barriers, and future plans. Sex Transm Dis.

[pmed-0050213-b018] Klausner JD, Wolf W, Fischer-Ponce L, Zolt I, Katz MH (2000). Tracing a syphilis outbreak through cyberspace. JAMA.

[pmed-0050213-b019] Curioso WH, Blas MM, Nodell B, Alva IE, Kurth AE (2007). Opportunities for providing Web-based interventions to prevent sexually transmitted infections in Peru. PLoS Med.

